# Differential Drug Survival of Second-Line Biologic Therapies in Patients with Psoriasis: Observational Cohort Study from the British Association of Dermatologists Biologic Interventions Register (BADBIR)

**DOI:** 10.1016/j.jid.2017.09.044

**Published:** 2018-04

**Authors:** Ireny Y.K. Iskandar, Richard B. Warren, Mark Lunt, Kayleigh J. Mason, Ian Evans, Kathleen McElhone, Catherine H. Smith, Nick J. Reynolds, Darren M. Ashcroft, Christopher E.M. Griffiths, BADBIR Study Group

**Affiliations:** 1 Centre for Pharmacoepidemiology and Drug Safety, Division of Pharmacy and Optometry, School of Health Sciences, Faculty of Biology, Medicine and Health, The University of Manchester, Manchester, UK; 2 Dermatology Centre, Salford Royal NHS Foundation Trust, The University of Manchester, Manchester Academic Health Science Centre, Manchester, UK; 3 Division of Musculoskeletal and Dermatological Sciences, School of Biological Sciences and NIHR Manchester Biomedical Research Centre, Faculty of Biology, Medicine and Health, The University of Manchester, Manchester, UK; 4 Arthritis Research UK Epidemiology Unit, Centre for Musculoskeletal Research, Manchester Academic Health Science Centre, The University of Manchester, Manchester, UK; 5 St. John’s Institute of Dermatology, Guy’s and St Thomas’ NHS Foundation Trust, London, UK; 6 Institute of Cellular Medicine, Medical School, Newcastle University, NIHR Newcastle Biomedical Research Centre Newcastle upon Tyne, UK; 7 Department of Dermatology, Royal Victoria Infirmary, Newcastle Hospitals NHS Foundation Trust, Newcastle upon Tyne, UK

**Keywords:** AE, adverse event, BADBIR, British Association of Dermatologists Biologic Interventions Register, CI, confidence interval, HR, hazard ratio, PASI, Psoriasis Area and Severity Index, TNFI, tumor necrosis factor inhibitors

## Abstract

Little is known about the drug survival of second-line biologic therapies for psoriasis in routine clinical practice. We assessed drug survival of second-line biologic therapies and estimated the risk of recurrent discontinuation due to adverse events or ineffectiveness in patients with psoriasis who had failed a first biologic therapy and switched to a second in a large, multicenter pharmacovigilance registry (n = 1,239; adalimumab, n = 538; etanercept, n = 104; ustekinumab, n = 597). The overall drug survival rate in the first year after switching was 77% (95% confidence interval = 74–79%), falling to 58% (55–61%) in the third year. Female sex, multiple comorbidities, concomitant therapy with cyclosporine, and a high Psoriasis Area and Severity Index at switching to the second-line biologic therapy were predictors of overall discontinuation (multivariable Cox proportional hazard model). Compared to adalimumab, patients receiving etanercept were more likely to discontinue therapy (hazard ratio = 1.87, 95% confidence interval = 1.24–2.83), whereas patients receiving ustekinumab were more likely to persist (hazard ratio = 0.46; 95% confidence interval = 0.33–0.64). Discontinuation of the first biologic therapy because of adverse events was associated with an increased rate of second drug discontinuation because of adverse events (hazard ratio = 2.55; 95% confidence interval = 1.50–4.32). In conclusion, drug survival rates differed among biologic therapies and decreased over time; second-line discontinuation because of adverse events was more common among those who discontinued first-line treatment for this reason. The results of this study should support clinical decision making when choosing second-line biologic therapy for patients with psoriasis.

## Introduction

Biologic therapies have markedly improved the management of moderate to severe psoriasis. The efficacy of these therapies has been established in large randomized clinical trials, with up to 88% of patients achieving at least a 75% improvement in the Psoriasis Area and Severity Index (PASI) (Nast et al., 2015, Reich et al., 2012). In addition, several prospective cohort studies have also shown the effectiveness of these therapies in routine clinical practice (Iskandar et al., 2017b, Norlin et al., 2012, Strober et al., 2016, Zweegers et al., 2016a).

Despite these impressive findings, approximately 11–35% of patients fail their first biologic therapy during the first year of treatment, either because of ineffectiveness or following the development of adverse events (AEs) (Warren et al., 2015). Switching biologic therapies on treatment failure is common (Iskandar et al., 2017a, Leman and Burden, 2012, Norlin et al., 2012), with several studies suggesting that initiating therapy with a second biologic is beneficial (Clemmensen et al., 2011, Downs, 2010, Gottlieb et al., 2012, Lecluse et al., 2009, Mazzotta et al., 2009, Ortonne et al., 2011, Van Lümig et al., 2010). However, to date, these studies have included relatively small numbers of patients (range = 10–282 patients), which makes it difficult to establish a substantive estimate of the risk of recurrent discontinuation because of AEs or ineffectiveness. Furthermore, the optimal choice of the subsequent treatment in those patients who fail or who are intolerant of the first-line biologic treatment is not established (Mauskopf et al., 2014).

Drug survival is a comprehensive measure of drug effectiveness, safety, and real-world utility (van den Reek et al., 2015a). Several studies reported on drug survival with biologic therapies among patients previously exposed to biologic therapies. Four of these studies have reported only on drug survival with tumor necrosis factor inhibitors (TNFIs) (Brunasso et al., 2012, Gniadecki et al., 2011, Inzinger et al., 2016, Menting et al., 2014), two studies involved the Danish National Psoriasis Biologic Safety Registry Data (Gniadecki et al., 2011, Gniadecki et al., 2015), one study involved the PSOriasis Longitudinal Assessment and Registry (Menter et al., 2016), and the other studies reported data from either a single or a limited number of dermatology centers (López-Ferrer et al., 2013, Umezawa et al., 2013, van den Reek et al., 2015b, van den Reek et al., 2014a, Zweegers et al., 2016b). The findings from these studies differ markedly; for instance, Menting et al. (2014) reported that drug survival did not differ significantly between biologic therapies among patients previously exposed to biologic therapies, whereas Gniadecki et al. (2015) found that the survival of ustekinumab was equal to that of adalimumab and infliximab but superior to that of etanercept. More recently, Menter et al. (2016) found that ustekinumab had superior drug survival compared with infliximab, adalimumab, and etanercept. Moreover, none of these studies took into consideration that the threshold for drug discontinuation may change over time (Dávila-Seijo et al., 2016) or investigated whether the reason for failing the first-line biologic therapy is predictive of the clinical outcome in patients receiving a second biologic therapy. Furthermore, Menter et al. (2016) included patients who could have discontinued their previous biologic therapy before enrollment into the register; this has the potential to introduce a source of bias due to left censorship.

Therefore, a number of clinically important questions remain unanswered. First, drug survival with second-line biologic therapies in routine clinical practice needs further exploration. In doing so, the effect of the increasing number of biologic therapies available to treat psoriasis in recent years and their effect on the threshold for drug discontinuation need to be considered (Dávila-Seijo et al., 2016). In addition, the risk of recurrent discontinuation because of AEs or ineffectiveness and whether the reason for failing a first biologic therapy is predictive of failure of a second is unknown and warrants investigation.

The British Association of Dermatologists Biologic Interventions Register (BADBIR) is a UK and Republic of Ireland prospective, longitudinal pharmacovigilance register. This represents an ideal resource to assess real-world drug survival with second-line biologic therapies for psoriasis because of its large size, a rigorous data collection process, inclusion of clinically relevant covariates, and high external validity through the participation of 153 dermatology centers (Burden et al., 2012). In this cohort study, we examined the comparative drug survival with second-line use of adalimumab, etanercept, and ustekinumab and identified clinically relevant risk factors for drug discontinuation. We also estimated the risk of recurrent discontinuation because of AEs or ineffectiveness.

## Results

**Figure 1:**
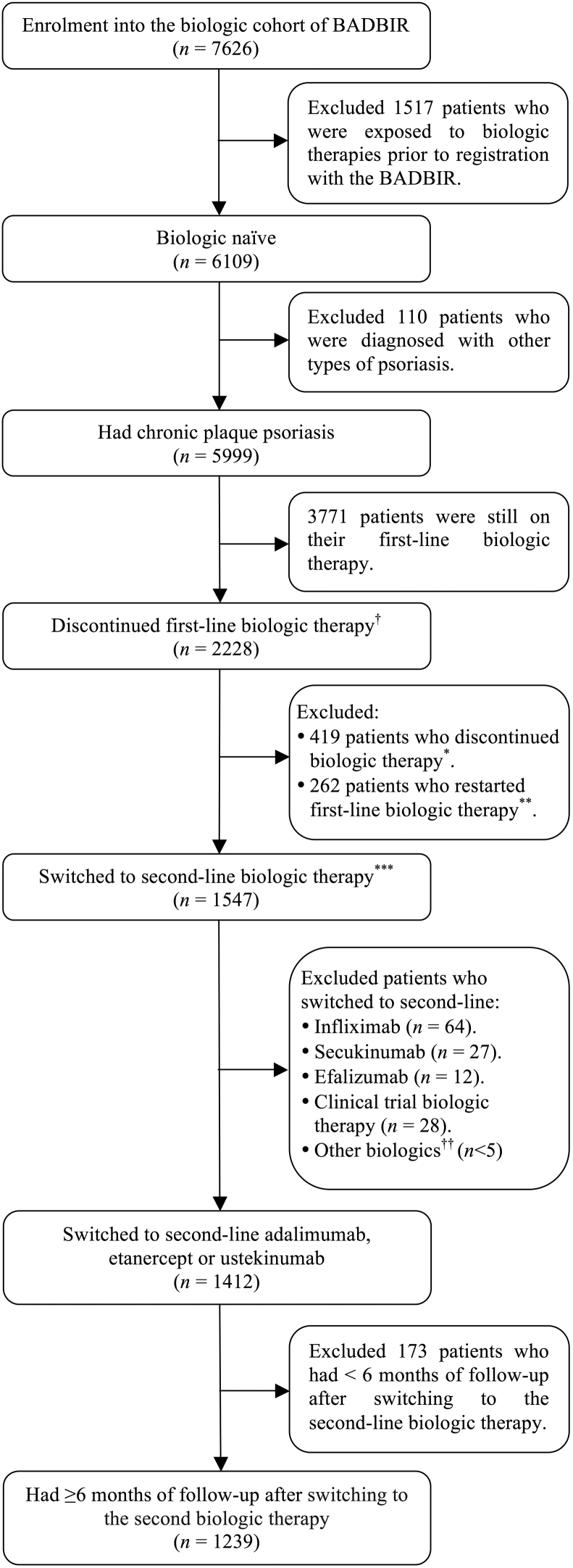
**Patient selection.**^†^Patients with a gap of 90 days or greater after the start date of the first-line biologic therapy were defined as discontinuing their first-line biologic therapy and were further classified into one of three mutually exclusive groups based on the treatment patterns after the first 90-day gap: discontinued, restarted, or switched therapy. ^∗^Patients were classified as *discontinued therapy* if they did not receive any biologic therapy after the first 90-day gap. ^∗∗^ Patients were classified as *restarted therapy* if they had a treatment gap that exceeded the 90-day period and subsequently restarted the same biologic therapy. ^∗∗∗^ Patients were classified as *switched therapy* if they initiated a new biologic therapy after the first 90-day gap (Iskandar et al., 2017a). ^††^Includes rituximab, certolizumab, or golimumab. BADBIR, British Association of Dermatologists Biologic Interventions Register.

From a prospective cohort of 6,109 biologic-naïve patients with psoriasis, we identified a total of 1,239 (adalimumab, n = 538; etanercept, n = 104; ustekinumab, n = 597) who failed their first biologic therapy and were switched to a second while under follow-up in the BADBIR (Figure 1). Overall, 1,181 (95%) of these patients failed first-line TNFIs, and 47 (4%) and 11 (1%) patients failed first-line ustekinumab or other biologic therapies, respectively (see Supplementary Table S1 online). Patients who failed first-line TNFIs were switched to second-line ustekinumab (50%), adalimumab (42%), and etanercept (8%); 89% and 46% of patients failing first-line ustekinumab and other biologic therapies were switched to second-line adalimumab and etanercept, respectively (see Supplementary Table S1). In total, 941 (76%) of those patients who were switched to second-line biologic therapies discontinued the first biologic therapy because of ineffectiveness, whereas 154 (12%) and 144 (12%) patients discontinued the first biologic therapy because of the development of AEs or for other reasons, respectively (see Supplementary Table S2 online).

At the time of switching to a second biologic therapy, the mean ± standard deviation age of patients was 46.3 ± 12.8 years, with 42% female. The mean PASI and Dermatology Life Quality Index were 12.4 ± 9.8 and 13.3 ± 13.7, respectively. Overall, 285 (23%) patients reported having psoriatic arthritis (PsA), and 70% reported having one or more comorbidities other than PsA. Baseline (at the time of switching) demographic and disease characteristics are summarized in Table 1.

**Table 1:** Demographic and disease characteristics at the time of switch to second biologic therapy

Characteristics	All Patients^1^ (*n=* 1239)	Etanercept (*n=* 104;8.4%)	Adalimumab (*n=*538; 43.4%)	Ustekinumab (*n=*597; 48.2%)
Demographic				
Age in years, mean (SD)	46.3 (12.8)	46.8 (12.2)	46.1 (12.7)	46.4 (13.0)
Female, n (%)	515 (41.6)	55 (52.9)	222 (41.3)	238 (39.9)
BMI^1^, n (%)				
Nonobese (<30 kg/m^2^)	525 (42.4)	41 (39.1)	229 (42.5)	255 (42.8)
Obese (≥30 kg/m^2^)	714 (57.6)	63 (60.9)	309 (57.5)	342 (57.2)
Smoking status^1^,^2^, n (%)				
Never smoked	393 (31.7)	26 (24.5)	190 (35.2)	178 (29.9)
Ex-smoker	404 (32.6)	33 (31.8)	168 (31.3)	203 (34.0)
Current smoker	442 (35.6)	45 (43.7)	180 (33.5)	216 (36.1)
Psoriatic arthritis, n (%)	285 (23.0)	32 (30.8)	130 (24.2)	123 (20.6)
Total number of co-morbidities (excluding PsA)^2^, n (%)				
No comorbidities	369 (29.8)	26 (25.0)	153 (28.4)	190 (31.8)
1–2 comorbidities	591 (47.7)	51 (49.0)	277 (51.5)	263 (44.1)
3–4 comorbidities	210 (17.0)	13 (12.5)	87 (16.2)	110 (18.4)
≥5 comorbidities	69 (5.6)	14 (13.5)	21 (3.9)	34 (5.7)
Disease, mean (SD)				
Disease duration in years^1^	22.9 (12.7)	22.9 (13.4)	23.3 (12.6)	22.5 (12.7)
Age of onset in years^1^	23.4 (13.4)	23.9 (12.8)	22.8 (13.2)	23.9 (13.7)
PASI^1^	12.4 (9.8)	12.9 (9.4)	11.3 (8.8)	13.4 (10.4)
DLQI^1^	13.3 (13.7)	14.2 (10.8)	12.5 (12.8)	13.9 (13.2)
Medication history, n (%)				
Concomitant methotrexate	130 (10.5)	12 (11.5)	63 (11.7)	55 (9.2)
Concomitant cyclosporine	67 (5.4)	4 (3.8)	26 (4.8)	37 (6.2)
Concomitant other systemics^3^	33 (2.7)	<5 (1.0)	16 (3.0)	16 (2.7)

Abbreviations: BMI, body mass index; DLQI, dermatology life quality index; PASI, Psoriasis Area and Severity Index; PsA, psoriatic arthritis; SD, standard deviation.

1 A multiple imputation model of 80 cycles was performed to account for missing data.

2 Collected only at the time of registration.

3 Includes any of acitretin, fumaric acid esters, hydroxcarbamide, azathioprine, and mycophenolate mofetil.

### Drug survival with second-line biologic therapies

Drug survival data for second-line biologic therapies were available for a mean ± standard deviation, total follow-up, and range of follow-up time of 2.7 ± 1.6; 2,405.7; and 0.5–7.8 person-years, respectively, with a mean ± standard deviation follow-up time for patients receiving adalimumab of 3.2 ± 1.7 years, those receiving etanercept of 2.7 ± 1.6 years, and those receiving ustekinumab of 2.3±1.3 years. Over the time frame of the study, 457 of 1,239 patients (37%) discontinued their second biologic therapy.

**Figure 2:**
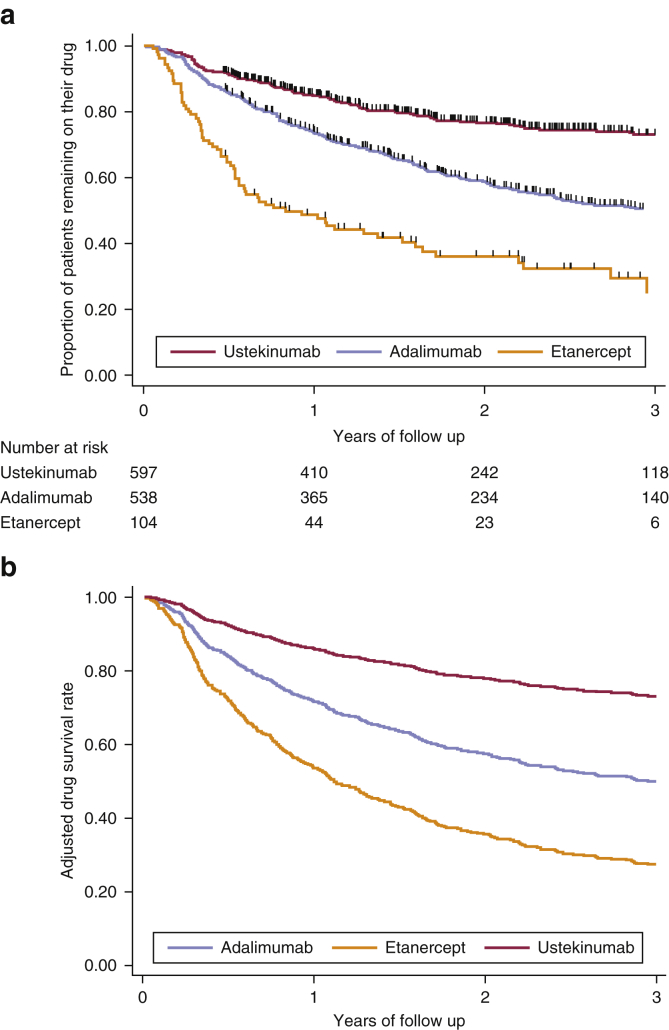
**Crude and adjusted drug survival curves of the second biologic course.** (**a**) Crude drug survival of the second biologic course showing disaggregated biologic data (Kaplan-Meier survival curve). (**b**) Adjusted drug survival curves using disaggregated data based on the overall multivariable Cox proportional hazard model in Table 3.

Kaplan-Meier survival analyses (Table 2) found an overall survival rate of 77% (95% confidence interval [CI] = 74–79%) one year after switching, falling to 58% (55–61%) at 3 years. For individual biologic therapies, the 1-year survival rate for ustekinumab was 85% (82–87%), for adalimumab was 74% (70–77%), and for etanercept was 49% (39–58%), falling to 73% (68–77%), 50% (46–55%), and 25% (14–37%), respectively, at 3 years (Figure 2a). One year after starting therapy with second-line biologic therapies, 15% (13–17%) of patients discontinued therapy because of ineffectiveness, 5% (4–7%) because of AEs, and 3% (2–4%) for other reasons (Table 2). The most common AEs to cause discontinuation of the second biologic therapy were infections (2%), nervous system disorders (1%), and skin and subcutaneous tissue disorders (1%). Supplementary Figure S1 online shows second-line drug survival for biologic therapies by discontinuation because of ineffectiveness or AEs.

**Table 2:** The overall and differential second-line biologic survival functions, stratified by reason for drug discontinuation, at years 1, 2 and 3^1^

Reasons for Drug Discontinuation	Second Biologic (n = 1,239)	Adalimumab (n = 538)	Etanercept (n = 104)	Ustekinumab (n = 597)
All reasons				
Year 1	0.77 (0.74–0.79)	0.74 (0.70–0.77)	0.49 (0.39–0.58)	0.85 (0.82–0.87)
Year 2	0.65 (0.62–0.68)	0.59 (0.54–0.63)	0.36 (0.26–0.46)	0.77 (0.73–0.80)
Year 3	0.58 (0.55–0.61)	0.50 (0.46–0.55)	0.25 (0.14–0.37)	0.73 (0.68–0.77)
Ineffectiveness				
Year 1	0.85 (0.83–0.87)	0.84 (0.80–0.87)	0.58 (0.47–0.68)	0.91 (0.88–0.93)
Year 2	0.78 (0.75–0.81)	0.74 (0.70–0.78)	0.47 (0.36–0.58)	0.87 (0.84–0.90)
Year 3	0.75 (0.72–0.78)	0.71 (0.66–0.75)	0.38 (0.23–0.53)	0.85 (0.81–0.88)
Adverse events				
Year 1	0.95 (0.93–0.96)	0.94 (0.91–0.95)	0.87 (0.78–0.92)	0.97 (0.95–0.98)
Year 2	0.92 (0.90–0.94)	0.91 (0.87–0.93)	0.85 (0.75–0.91)	0.95 (0.92–0.97)
Year 3	0.89 (0.86–0.91)	0.85 (0.81–0.89)	0.73 (0.51–0.86)	0.94 (0.92–0.96)

1 Data presented as mean (95% confidence interval).

A sensitivity analysis investigating the impact of switching from first-line TNFIs showed similar findings to the overall patient cohort. The corresponding overall survival rate was 78% (75–80%) 1 year after switching, falling to 59% (56–63%) at 3 years (see Supplementary Table S3 online). For individual biologic therapies, the 1-year survival rate for ustekinumab was 85% (81–87%), for adalimumab was 75% (71–79%), and for etanercept was 46% (36–56%), falling to 73% (68–77%), 51% (46–56%), and 33% (22–43%), respectively, at 3 years (see Supplementary Table S3). Other sensitivity analyses investigating the impact of switching from first-line etanercept (see Supplementary Table S4 and Supplementary Figure S2a online) or adalimumab (see Supplementary Table S5 and Supplementary Figure S2b online) to second-line biologic therapies also found that the differential overall drug survival with second-line biologic therapies was similar to the findings for the overall patient cohort.

### Predictors of drug survival with second-line biologic therapies

Table 3 presents results from the univariable and multivariable analyses examining predictors of overall drug discontinuation, discontinuation due to ineffectiveness, and discontinuation due to AEs.

**Table 3:** Univariate and multivariate Cox proportional hazard analyses for drug discontinuation, presented by reason for discontinuation from second-line biologic therapy

Variable	Univariate and Multivariate Cox Proportional Hazard Analysis for Drug Discontinuation					
	Overall Discontinuation		Discontinuation due to Ineffectiveness		Discontinuation due to Adverse Events	
	Univariate	Multivariate	Univariate	Multivariate	Univariate	Multivariate
Demographics						
Age^1^	0.97 (0.90–1.04)	0.95 (0.87–1.04)	1.01 (0.91–1.11)	0.97 (0.86–1.10)	1.01 (0.87–1.18)	1.05 (0.88–1.26)
Female	**1.38 (1.15–1.66)**^2^	**1.31 (1.08–1.60)**	**1.34 (1.05–1.72)**	1.22 (0.94–1.59)	**1.55 (1.05–2.30)**	**1.53 (1.01–2.32)**
Obesity status^3^						
Obese (BMI ≥ 30 kg/m^2^)	1.26 (0.99–1.61)	1.22 (0.94–1.58)	1.35 (0.96–1.92)	1.29 (0.90–1.86)	0.94 (0.55–1.61)	0.89 (0.51–1.55)
Smoking status^4^						
Ex-smoker	0.84 (0.65–1.08)	0.85 (0.66–1.10)	0.93 (0.66–1.30)	0.93 (0.66–1.32)	0.81 (0.46–1.44)	0.84 (0.46–1.51)
Current smoker	0.96 (0.75–1.23)	0.95 (0.73–1.23)	0.99 (0.70–1.39)	0.97 (0.68–1.38)	0.90 (0.52–1.54)	0.87 (0.50–1.54)
Comorbidities^5^						
Psoriatic arthritis	1.03 (0.83–1.28)	0.86 (0.68–1.09)	1.16 (0.88–1.55)	0.92 (0.68–1.25)	0.91 (0.56–1.47)	0.79 (0.47–1.32)
1–2 comorbidities	1.01 (0.81–1.26)	0.97 (0.77–1.24)	1.02 (0.76–1.39)	0.94 (0.68–1.30)	1.11 (0.68–1.81)	1.04 (0.62–1.77)
3–4 comorbidities	**1.35 (1.03–1.77)**	**1.46 (1.07–1.98)**	1.43 (0.99–2.06)	1.40 (0.93–2.11)	1.74 (0.98–3.08)	**1.95 (1.02–3.73)**
≥5 comorbidities	**1.58 (1.08–2.32)**	**1.56 (1.02–2.39)**	1.58 (0.94–2.66)	1.19 (0.66–2.13)	1.37 (0.56–3.36)	1.52 (0.57–4.06)
Disease						
Disease duration^1^	0.95 (0.88–1.03)	0.95 (0.87–1.03)	0.99 (0.90–1.10)	0.99 (0.88–1.12)	0.87 (0.73–1.03)	**0.79 (0.66–0.95)**
PASI	1.01 (0.99–1.03)	**1.02 (1.01–1.04)**	1.02 (0.99–1.03)	1.02 (0.99–1.04)	1.00 (0.97–1.03)	1.02 (0.98–1.06)
DLQI	0.99 (0.97–1.01)	0.98 (0.96–1.00)	1.00 (0.98–1.02)	0.99 (0.97–1.02)	0.99 (0.96–1.03)	0.98 (0.94–1.03)
Concomitant^6^ methotrexate	1.23 (0.94–1.62)	1.01 (0.76–1.35)	**1.66 (1.19–2.31)**	1.33 (0.93–1.90)	**0.34 (0.12–0.91)**	**0.29 (0.11–0.81)**
Concomitant^6^ cyclosporine	**1.74 (1.17–2.58)**	**1.55 (1.02–2.36)**	**2.00 (1.20–3.31)**	**2.17 (1.28–3.68)**	1.45 (0.59–3.56)	0.93 (0.34–2.58)
First-line biologic therapy^7^						
Adalimumab	0.82 (0.67–1.00)	1.07 (0.75–1.52)	0.92 (0.70–1.21)	1.18 (0.72–1.95)	0.70 (0.44–1.10)	0.98 (0.45–2.14)
Infliximab	0.97 (0.63–1.50)	1.02 (0.63–1.64)	1.31 (0.77–2.23)	1.54 (0.85–2.79)	0.60 (0.19–1.93)	0.60 (0.17–2.09)
Ustekinumab	**1.85 (1.23–2.77)**	1.51 (0.97–2.35)	**1.87 (1.08–3.25)**	1.38 (0.76–2.51)	**2.24 (1.02–4.93)**	1.66 (0.71–3.87)
Other biologics^8^	1.10 (0.49–2.49)	0.87 (0.35–2.18)	—	—	**4.26 (1.68–10.80)**	**4.62 (1.28–16.62)**
Second-line biologic therapy^9^						
Etanercept	**2.13 (1.63–2.79)**	**1.87 (1.24–2.83)**	**2.77 (1.98–3.87)**	**2.44 (1.40–4.25)**	**1.99 (1.12–3.52)**	1.64 (0.67–4.01)
Ustekinumab	**0.50 (0.41–0.62)**	**0.46 (0.33–0.64)**	**0.47 (0.35–0.63)**	**0.41 (0.26–0.64)**	**0.42 (0.27–0.67)**	**0.38 (0.18–0.77)**
Reason for discontinuing first biologic therapy^10^						
Ineffectiveness	—	—	1.08 (0.80–1.46)	1.04 (0.69–1.56)	—	—
Adverse events^11^	1.27 (0.97–1.66)	**1.34 (1.01–1.78)**	—	—	**2.09 (1.28–3.41)**	**2.55 (1.50–4.32)**
Other	1.07 (0.80–1.42)	1.09 (0.80–1.47)	0.83 (0.55–1.27)	0.83 (0.48–1.43)	0.97 (0.50–1.89)	0.82 (0.38–1.75)
Drug year^12^	**0.93 (0.88–0.98)**	0.98 (0.91–1.04)	0.94 (0.87–1.02)	0.99 (0.91–1.08)	0.90 (0.80–1.02)	0.98 (0.85–1.13)

Abbreviations: BMI, body mass index; DLQI, Dermatology Life Quality Index; PASI, Psoriasis Area and Severity Index.

Data presented as hazard ratio (95% confidence interval).

1 To evaluate the hazard ratio for every 10-year increase in age and disease duration at the time of switching to the second-line biologic therapy, continuous variables of age and disease duration were transformed to age and disease duration divided by 10.

2 *P* < 0.05, shown in bold.

3 Reference category: nonobese (BMI < 30kg/m^2^).

4 Reference category: never smoker.

5 Reference category: no comorbidities (excluding psoriatic arthritis). Includes (according to Medical Dictionary for Regulatory Activities system organ class): blood and lymphatic system disorders; cardiac disorders; congenital, familial, and genetic disorders; ear and labyrinth disorders; endocrine disorders; eye disorders; gastrointestinal disorders; general disorders and administration site conditions; hepatobiliary disorders; immune system disorders; infections and infestations; injury, poisoning, and procedural complications; investigations; metabolism and nutrition disorders; musculoskeletal and connective tissue disorders; neoplasms benign, malignant, and unspecified; nervous system disorders; pregnancy, puerperium and perinatal conditions; psychiatric disorders; renal and urinary disorders; reproductive system and breast disorders; respiratory, thoracic and mediastinal disorders; skin and subcutaneous tissue disorders; Social circumstances; surgical and medical procedures; vascular disorders.

6 Time-varying covariates.

7 Reference category: etanercept.

8 Includes efalizumab and clinical trial biologic therapies.

9 Reference category: adalimumab.

10 Reference category: ineffectiveness.

11 For the multivariate analysis examining predictors for withdrawal due to ineffectiveness, adverse events was used as a reference category.

12 Drug year (calendar year the second-line biologic therapy was prescribed) is adjusted for in the multivariate analysis.

For overall discontinuation of the second biologic therapy, the multivariable model showed that being female (hazard ratio [HR] = 1.31, 95% confidence interval [CI] = 1.08–1.60), having multiple comorbidities other than PsA compared with having no comorbidities (three or four comorbidities: HR = 1.46, 95% CI = 1.07–1.98; five or more comorbidities: HR = 1.56, 95% CI = 1.02–2.39), having a higher PASI at the time of switching to the second biologic therapy (per 1-point increase in PASI score: HR = 1.02, 95% CI = 1.01–1.04), concomitantly using cyclosporine with the second biologic therapy (HR = 1.55, 95% CI = 1.02–2.36), and taking etanercept rather than adalimumab (HR = 1.87, 95% CI = 1.24–2.83) were predictors of discontinuation. In contrast, taking ustekinumab rather than adalimumab (HR = 0.46, 95% CI = 0.33–0.64) was a predictor for drug survival (Table 3 and Figure 2b). Furthermore, patients who discontinued their first biologic therapy because of development of an AE were associated with significantly higher overall discontinuation rates of the second biologic therapy compared with patients who discontinued their first biologic therapy because of ineffectiveness (HR = 1.34, 95% CI = 1.01–1.78).

For discontinuation of the second biologic therapy because of ineffectiveness, concomitantly using cyclosporine with the second biologic therapy (HR = 2.17, 95% CI = 1.28–3.68) and taking etanercept rather than adalimumab (HR = 2.44, 95% CI = 1.40–4.25) were predictors of discontinuation, whereas taking ustekinumab rather than adalimumab (HR = 0.41, 95% CI = 0.26–0.64) was a predictor for drug survival. There was no significantly increased risk of drug discontinuation because of ineffectiveness in those patients who discontinued their first biologic therapy for this reason (HR = 1.04, 95% CI = 0.69–1.56).

For discontinuation of the second biologic therapy because of AEs, female sex (HR = 1.53, 95% CI = 1.01–2.32), the presence of multiple comorbidities other than PsA compared with having no comorbidities (three or four comorbidities: HR = 1.95, 95% CI = 1.02–3.73), and having other biologic therapies (such as efalizumab and clinical trial biologic therapies) rather than etanercept as first-line biologic therapies (HR = 4.62, 95% CI = 1.28–16.62) were predictors of discontinuation, whereas having a longer disease duration at the time of switching to the second biologic therapy (per 10 years increase in disease duration: HR = 0.79, 95% CI = 0.66–0.95), concomitantly using methotrexate with the second biologic therapy (HR = 0.29, 95% CI = 0.11–0.81), and taking ustekinumab rather than adalimumab (HR = 0.38, 95% CI = 0.18–0.77) were predictors for drug survival. First drug discontinuation due to AEs was associated with an increased rate of second drug discontinuation due to this reason (HR = 2.55, 95% CI = 1.50–4.32). However, only three patients experienced the same AE during therapy with the first and second biologic courses (skin and subcutaneous tissue disorder, n = 1; infections, n = 2).

## Discussion

In this large prospective cohort study, we found that after failure of a first-line biologic therapy, most patients with psoriasis who were switched to a second biologic therapy continued with this therapy for an estimated 1-year drug survival rate of 77%. This is similar to the estimated 1-year drug survival rates of first-line biologic therapies, which we have reported on previously (Warren et al., 2015). One of the most notable findings was that the likelihood of patients experiencing an AE with the second agent was increased approximately 2.5-fold if the first agent was also stopped because of an AE, although recurrence of the same AE was rare. The mechanisms that may underlie this require further investigation.

Compared with adalimumab, taking etanercept was a predictor for discontinuation overall and due to ineffectiveness, whereas taking ustekinumab was a predictor of drug survival. This difference persisted after adjustment for important clinical factors likely to influence treatment response. Other important predictors for drug discontinuation included female sex, multiple comorbidities, a high PASI at the time of switching to the second biologic therapy and concomitantly using cyclosporine with the second biologic therapy.

### Comparisons with existing literature

Consistent with some, but not all, previous studies, we found that drug survival rates for the first and second courses of biologic therapies were comparable (Menting et al., 2014, van den Reek et al., 2014b). However, compared with those studies, our research has important strengths: our sample size was much larger, thereby giving the study more power for the investigation of comparative second-line biologic survival, and we included patients from multiple dermatology centers, thus ensuring high external validity.

Our finding that ustekinumab had better drug survival rate compared with TNFIs among patients who switched to second-line biologic therapies is in line with results reported by Menter et al. (2016). However, in contrast to our findings, Gniadecki et al. (2015), using the Danish National Psoriasis Biologic Safety Registry Data, reported that the survival of ustekinumab was equal to that of adalimumab but was superior to that of etanercept among patients previously exposed to biologic therapies. However, the smaller sample size of this earlier study (only 576 patients) is likely to have limited its power to investigate comparative biologic therapy survival. Furthermore, Gniadecki et al. (2015) did not adjust for factors that could influence second-line biologic drug survival. Such factors include patients’ demographic and disease characteristics; the presence of PsA and other comorbidities; the concomitant prescription of methotrexate and cyclosporine; and reason for discontinuation of the first biologic therapy, which we were able to take account of in this study.

### Factors associated with biologic survival

Discontinuation of second-line biologic therapies was associated with a range of factors, including choice of biologic therapy, patient demographic characteristics, and disease-related factors. We have shown that when compared with adalimumab, taking etanercept was a predictor for discontinuation overall and because of ineffectiveness, whereas taking ustekinumab was a predictor of drug survival. Possible reasons contributing to the observed higher drug survival with second-line use of ustekinumab could include differences in clinical effectiveness (Griffiths et al., 2010, Reich et al., 2012), speed of onset of action (Nast et al., 2013), low immunogenicity (Carrascosa et al., 2014), and nurse-administered injections provided every 12-weeks, compared with the more frequent self-injection regimens for other biologic therapies, which may contribute to improved drug adherence and treatment satisfaction (Goren et al., 2016, Hsu and Gniadecki, 2016, Schaarschmidt et al., 2015).

Our study also found that patients were more likely to discontinue second-line biologic therapy because of an AE if the first-line biologic therapy was also stopped for the same reason. However, discontinuation due to lack of effectiveness of a first biologic therapy did not appear to predict discontinuation due to ineffectiveness of a second. Hence, patients and clinicians should be reassured that ineffectiveness of the first biologic therapy does not necessarily mean that there would be a greater likelihood of experiencing ineffectiveness with the second biologic therapy, but it is critically important to remain vigilant for AEs, particularly among those patients who discontinued their first biologic therapy because of an AE. To the best of our knowledge, the magnitude of the effect that the reason for failing the first-line biologic therapy has on the clinical outcome in patients with psoriasis receiving a second biologic therapy has not been previously reported. Hence, future studies are required to validate our findings. An earlier study of the rheumatoid arthritis cohort within the British Society for Rheumatology Biologics Register also found that the likelihood of recurrent discontinuation due to AE with the second biologic therapy was increased by more than 2-fold if the first biologic therapy was discontinued because of an AE (Hyrich et al., 2007).

Existing data and guidelines for treatment sequencing after failure of first-line biologic therapies are limited. Our study found that the drug survival rate of second-line biologic therapies was comparable to those reported previously for first-line biologic therapies, thus supporting findings from previous studies that switching therapies is a viable option (Leman and Burden, 2012, Norlin et al., 2012). These findings provide valuable data to inform cost-effectiveness analyses of sequential use of different biologic therapies in patients with psoriasis, because many existing cost-effectiveness models are limited by not considering subsequent treatment regimens (Mauskopf et al., 2014). Switching to another class of biologic therapy is also useful, as shown in our study by the high drug survival rates of second-line ustekinumab among patients for whom first-line TNFIs failed. However, we had a very small cohort of 47 patients for whom first-line ustekinumab failed and who were switched to second-line TNFIs. Therefore, future studies with a larger cohort of patients failing first-line ustekinumab will be required to establish the potential benefits of switching to second-line TNFIs.

Concomitantly using cyclosporine with the second biologic therapy was found to predict the likelihood of drug failure due to ineffectiveness. In contrast, concomitantly using methotrexate with the second biologic therapy was associated with lower risk of discontinuing the second biologic therapy because of AEs. However, these observations could potentially be attributed to confounding by indication for use. Combination therapies are likely to be used in patients who are not responding adequately to biologic therapy (Cather and Crowley, 2014).

Because the BADBIR was established primarily as a pharmacovigilance register, there are some limitations to studying differential drug survival of second-line biologic therapies that should be considered when interpreting our findings. First, information on patients’ adherence to treatment was not available. Furthermore, the influence of dose escalation on differential biologic drug survival was not assessed. However, we have shown previously that patients in the BADBIR routinely receive the recommended dosing regimen of biologic therapies but that concomitant treatment with other systemic therapies occurs commonly (Iskandar et al., 2017a). One particular challenge that we faced is that patients’ demographic characteristics were not re-recorded in the BADBIR at the time of switch from one biologic therapy to another. As a consequence, demographic characteristics were determined from the patients’ records at the time of registration with the BADBIR. This included data on smoking status and comorbidities. It is possible that some patients may have developed new comorbidities or changed smoking status during the time when they were receiving their first biologic therapy. An inherent limitation in any observational study is nonrandomization, which may introduce selection bias, and although this is partially negated by adjustment for clinically relevant covariates, the presence of other unmeasured confounders, such as the severity of PsA and its response to treatment, as well as the intention behind concomitant medication, cannot be determined. In the future, it will be important to examine the comparative drug survival of the recently approved anti-IL-17 biologic therapies (secukinumab and ixekizumab), and as longer-term follow-up of patients permits, then additional insights into drug survival with third-line and subsequent courses of biologic therapies could also be explored.

## Summary

This large prospective cohort study provides insights into the differential drug survival of second-line biologic therapies in routine clinical practice. We found that 77% of patients who were switched to a second biologic therapy continued to receive the new treatment for at least 12 months. This shows clearly that patients experiencing treatment failure with one biologic therapy can benefit from switching to another. However, second-line discontinuation due to AEs was more common among those who discontinued first-line treatment because of AEs. The results of this study should support clinical decision making when choosing second-line biologic therapy for patients with psoriasis.

## Materials and Methods

The BADBIR, established in September 2007, compares a cohort of patients with psoriasis receiving biologic therapies versus a similar cohort receiving conventional systemic therapies. Details about the design of the BADBIR and the disease characteristics of its participants have been published previously (Burden et al., 2012, Iskandar et al., 2015).

### Baseline data and follow-up

Baseline data were collected with patient consent and included patients’ demographic characteristics and lifestyle information: for example, smoking; details of type and severity of psoriasis and year of onset; standardized measures of health status using self-reported outcome measures (Dermatology Life Quality Index); detailed information about the patients’ current and previous treatment for psoriasis; and the patients’ comorbidities, the details of which were classified using the Medical Dictionary for Regulatory Activities system (Bousquet et al., 2005).

The BADBIR aimed to follow up with all patients at 6-month intervals for 3 years and then annually thereafter, even if the patient stopped or switched their therapy. Details of the biologic therapies, including any change in the therapy, gaps in treatment, start and stop dates, and reasons for discontinuation were recorded. The PASI and Dermatology Life Quality Index, along with their dates, were also documented. Information on any new concomitant systemic therapies for psoriasis and their start and stop dates were also captured. Details of the AEs were classified using the Medical Dictionary for Regulatory Activities system.

### Study population

Subjects in this study were selected from the April 2016 data cutoff. All patients with chronic plaque psoriasis who registered with the BADBIR as biologic-naïve patients; experienced treatment failure with their first biologic therapy for any reason; and then switched to second-line treatment with adalimumab, etanercept, or ustekinumab were eligible for inclusion in this analysis if they had one or more dermatologist follow-ups (i.e., with follow-up data of ≥6 months) after switching to the second biologic therapy, because data on drug persistence were otherwise not available. Patients who were switched to a second biologic therapy were subdivided into three groups based on whether they (i) did not show adequate response to the first biologic therapy, (ii) had developed an AE while receiving the first biologic therapy, or (iii) discontinued the first agent because of other reasons, for example, noncompliance.

### Statistical analysis

*Drug survival* was defined as “the length of time from initiation to discontinuation of therapy” (Cramer et al., 2008, pp 45). Discontinuation of therapy was defined as any gap in treatment for longer than 90 days, to disregard temporary treatment discontinuation during an infection or elective surgery and to take into account the early UK licensing prescription of etanercept in an intermittent dosing regimen with gaps of fewer than 90 days (Warren et al., 2015). The discontinuation date included the earliest date of any switches to third-line biologic therapy or death while registered on the BADBIR. This definition is in accordance with other drug survival studies in psoriasis and psoriatic arthritis (Esposito et al., 2013, Gniadecki et al., 2011, Saad et al., 2009, van den Reek et al., 2014a, Warren et al., 2015).

Differences in drug survival between second-line biologic therapies were examined using Kaplan-Meier survival analysis, with censorship occurring if a patient had not discontinued the biologic therapy at the last available follow-up date. Reasons for discontinuation, classified as due to ineffectiveness, due to AEs, and “other,” were noted for each biologic agent. Patients were categorized as having an AE if they either stopped therapy because of an AE or because of both an AE and ineffectiveness.

An a priori list of covariates was determined to address potential predictors of discontinuation (as presented in Table 3). Adalimumab was used as the reference biologic therapy with which the others were compared. Body mass index, derived from measurements of height and weight dated within 3 months before switching to the second biologic therapy, was categorized into a binary *obese*/*nonobese* variable. The baseline (switching-time) age and disease duration of the patients were calculated from patients’ ages recorded in their baseline questionnaires and the time of switching. Switching-time PASI and Dermatology Life Quality Index were identified if they were dated within 3 months before switching. Other demographic characteristics (that would not change by time) were obtained from the patients’ baseline forms and included sex and age of disease onset. Comorbidity with PsA was collected both at registration and follow-up, whereas other comorbidities and the patients’ smoking status were collected only at the time of registration. Concomitant methotrexate and cyclosporine were analyzed as time-varying covariates throughout the period of follow-up. The year of second biologic course prescription was included for adjustment to account for the fact that the available treatment options have changed over time.

Univariable and multivariable Cox proportional hazard models were used to identify factors associated with the second biologic course discontinuation. The proportional hazard assumption was tested formally using Schoenfeld residuals. Separate models were developed to analyze overall discontinuation, discontinuation due to ineffectiveness, and discontinuation due to AEs. In addition to determining the relationship between the reasons for stopping the first biologic therapy and the reasons for stopping the second biologic therapy, we undertook separate analyses stratified according to the reason for stopping the first biologic course.

To account for missing data, the details of which are listed in Supplementary Table S6 online, we generated 80 imputed datasets. In each dataset, missing values were replaced by values randomly selected from the expected distribution of that variable based on the measured and imputed values of all variables for that individual. This approach enables all subjects to be used in the analysis, avoiding the selection bias that would be likely if only subjects with complete data were analyzed (Bodner, 2008). In general, increasing the number of imputations reduces the width of any CIs, but there is a law of diminishing returns. We chose to generate 80 imputed datasets, because this number resulted in a relative efficiency for all parameters of 99% (i.e., we could reduce the width of the CIs by only 1% by increasing the number of imputations indefinitely). Sensitivity analyses were performed to investigate the differential drug survival with second-line biologic therapies stratified by the first-line biologic therapy the patient was switched from (see Supplementary Figure S2 and Supplementary Tables S3, S4, and S5). Analyses were performed using STATA version 14.0 (Stata Corp, College Station, TX).

### Ethical approval

The BADBIR was approved in March 2007 by NHS Research Ethics Committee North West England, reference 07/MRE08/9. All subjects gave written, informed consent for their participation in the registry.

## ORCID

Ireny YK Iskandar: http://orcid.org/0000-0002-8030-1908

## Conflict of Interest

DMA has received grant funding from AbbVie and served on advisory boards for Pfizer and GSK. RBW has acted as a consultant and/or speaker and/or received research grants for AbbVie, Amgen, Almirall, Celgene, Eli Lilly, Pfizer, Leo-Pharma, Novartis, Janssen, Medac, Xenoport. KJM received payment for developing and delivering educational presentations for Janssen-Cilag, Ltd., and Eli Lilly, Ltd. CHS’s department has received funding for research support from pharmaceutical companies that make biologic therapies including AbbVie, Janssen, Novartis, Wyeth and Pﬁzer. NJR has received honoraria, travel support, and/or research grants (Newcastle University) from AbbVie, Amgen, AstraZeneca, Bristol-Myers Squibb, Celgene, Genentech, Janssen, Leo-Pharma Research Foundation, Novartis, Pfizer, and Stiefel GSK. CEMG has received honoraria and/or research grants from AbbVie, Actelion, Almirall, Amgen, Celgene, Galderma, LEO Pharma, Eli Lilly, Stiefel GSK, Janssen, MSD, Novartis, Pfizer, Sandoz, Sun Pharmaceuticals, and UCB Pharma. The remaining authors state no conflict of interest.
