# Supplementary material for: Differential Drug Survival of Second-Line Biologic Therapies in Patients with Psoriasis: Observational Cohort Study from the British Association of Dermatologists Biologic Interventions Register (BADBIR)
Source: J Invest Dermatol. 2018 Apr;138(4):775–84. doi: 10.1016/j.jid.2017.09.044 (PMC5869053; doi:10.1016/j.jid.2017.09.044)
Supplement: Supplementary Tables S1–S6 and Supplementary Figures S1 and S2 [file mmc1.pdf]

Supplementary Material

Table S1: Patterns of biologic therapies switches

**First biologic therapy**	**Second biologic therapy** Adalimumab	Etanercept	Ustekinumab
**TNF Inhibitors (*n* = 1181)**	**492 (41.7%)**	**94 (7.9%)**	**595 (50.4%)**
Adalimumab	NA	88 (17.0%)	429 (83.0%)
Etanercept	473 (77.5%)	NA	137 (22.5%)
Infliximab	19 (35.2%)	6 (11.1%)	29 (53.7%)
**IL-12/23 Inhibitor (*n* = 47)**			
Ustekinumab	42 (89.4%)	5 (10.6%)	NA
**Other Biologics* (*n* = 11)**	<5	5 (45.5%)	<5

* Includes efalizumab and clinical trial biologic

Table S2: Patterns of biologic therapies switches, based on the reason for discontinuation of the first biologic therapy

**Reason for discontinuing 1st biologic therapy**	**Second biologic therapy**		
	Adalimumab	Etanercept	Ustekinumab
Total no. of patients on 2nd biologic therapy (*n* = 1239)	538 (43.4%)	104 (8.4%)	597 (48.2%)
Ineffectiveness (*n* = 941)	433 (46.0%)	67 (7.1%)	441 (46.9%)
AEs (*n* = 154)	46 (29.9%)	20 (13.0%)	88 (57.1%)
Other (*n* = 144)	59 (41.0%)	17 (11.8%)	68 (47.2%)

Table S3: The overall and differential second-line biologic survival functions among patients who switched from TNF inhibitors, stratified by reason for 2nd biologic drug discontinuation, at years 1, 2 and 3

Reasons for drug discontinuation	Second Biologic (n = 1181)	Adalimumab (n = 492)	Etanercept (n = 94)	Ustekinumab (n = 595)
All reasons				
Year 1	0.78 [0.75 - 0.80]	0.75 [0.71 - 0.79]	0.46 [0.36 - 0.56]	0.85 [0.81 - 0.87]
Year 2	0.66 [0.63 - 0.69]	0.60 [0.55 - 0.64]	0.35 [0.25 - 0.45]	0.77 [0.72 - 0.80]
Year 3	0.59 [0.56 - 0.63]	0.51 [0.46 - 0.56]	0.33 [0.22 - 0.43]	0.73 [0.68 - 0.77]
Ineffectiveness				
Year 1	0.86 [0.84 - 0.86]	0.85 [0.81 - 0.88]	0.56 [0.44 - 0.66]	0.91 [0.88 - 0.93]
Year 2	0.78 [0.76 - 0.81]	0.75 [0.70 - 0.79]	0.43 [0.31 - 0.55]	0.87 [0.84 - 0.90]
Year 3	0.76 [0.73 - 0.78]	0.71 [0.66 - 0.75]	0.43 [0.31 - 0.55]	0.85 [0.81 - 0.88]
Adverse events				
Year 1	0.95 [0.94 - 0.96]	0.94 [0.92 - 0.96]	0.87 [0.78 - 0.93]	0.97 [0.95 - 0.98]
Year 2	0.93 [0.91 - 0.94]	0.92 [0.88 - 0.94]	0.87 [0.78 - 0.93]	0.95 [0.92 - 0.97]
Year 3	0.90 [0.87 - 0.92]	0.86 [0.82 - 0.90]	0.81 [0.63 - 0.91]	0.94 [0.92 - 0.96]

Data presented as mean (95% confidence interval).

Table S4: The overall and differential second-line biologic survival functions among patients who switched from etanercept, stratified by reason for 2nd biologic drug discontinuation, at years 1, 2 and 3

Reasons for drug discontinuation	Second Biologic (n = 610)	Adalimumab (n = 473)	Ustekinumab (n = 137)
All reasons			
Year 1	0.78 [0.74 - 0.81]	0.75 [0.71 - 0.79]	0.87 [0.80 - 0.92]
Year 2	0.63 [0.59 - 0.67]	0.60 [0.55 - 0.64]	0.77 [0.68 - 0.83]
Year 3	0.55 [0.51 - 0.60]	0.51 [0.46 - 0.56]	0.71 [0.60 - 0.79]
Ineffectiveness			
Year 1	0.87 [0.84 - 0.90]	0.85 [0.81 - 0.88]	0.95 [0.89 - 0.97]
Year 2	0.78 [0.74 - 0.82]	0.75 [0.70 - 0.79]	0.89 [0.81 - 0.94]
Year 3	0.74 [0.70 - 0.78]	0.71 [0.66 - 0.76]	0.84 [0.74 - 0.90]
Adverse events			
Year 1	0.94 [0.93 - 0.96]	0.94 [0.91 - 0.96]	0.98 [0.93 - 0.99]
Year 2	0.92 [0.89 - 0.94]	0.91 [0.88 - 0.94]	0.94 [0.87 - 0.97]
Year 3	0.88 [0.84 - 0.91]	0.86 [0.82 - 0.90]	0.94 [0.87 - 0.97]

Data presented as mean (95% confidence interval).

Table S5: The overall and differential second-line biologic survival functions among patients who switched from adalimumab, stratified by reason for 2nd biologic drug discontinuation, at years 1, 2 and 3

Reasons for drug discontinuation	Second Biologic (n = 517)	Etanercept (n = 88)	Ustekinumab (n = 429)
All reasons			
Year 1	0.77 [0.73 - 0.81]	0.48 [0.38 - 0.58]	0.83 [0.79 - 0.87]
Year 2	0.70 [0.65 - 0.74]	0.36 [0.26 - 0.47]	0.77 [0.72 - 0.81]
Year 3	0.67 [0.62 - 0.71]	0.33 [0.22 - 0.45]	0.74 [0.69 - 0.78]
Ineffectiveness			
Year 1	0.85 [0.81 - 0.88]	0.58 [0.46 - 0.68]	0.90 [0.87 - 0.93]
Year 2	0.80 [0.75 - 0.83]	0.45 [0.32 - 0.56]	0.87 [0.83 - 0.90]
Year 3	0.79 [0.75 - 0.83]	0.45 [0.32 - 0.56]	0.86 [0.82 - 0.89]
Adverse events			
Year 1	0.95 [0.93 - 0.97]	0.88 [0.78 - 0.94]	0.97 [0.94 - 0.98]
Year 2	0.94 [0.91 - 0.96]	0.88 [0.78 - 0.94]	0.95 [0.92 - 0.97]
Year 3	0.93 [0.89 - 0.95]	0.81 [0.61 - 0.92]	0.94 [0.91 - 0.97]

Data presented as mean (95% confidence interval).

Table S6: Missing data

Variable	Number of patients with missing data	Percentage of missing data
Smoking status*	261	21.1%
Obesity category**	786	63.4%
PASI**	441	35.6%
DLQI**	797	64.3%
Disease duration***	11	0.9%

* at time of registration into BADBIR
** dated within 3 months prior to switching to 2nd biologic therapy
*** at time of switching to the 2nd biologic therapy

Figure S1: Crude drug survival of the second biologic course limited to discontinuation of the second biologic due to ineffectiveness or adverse events (AEs).

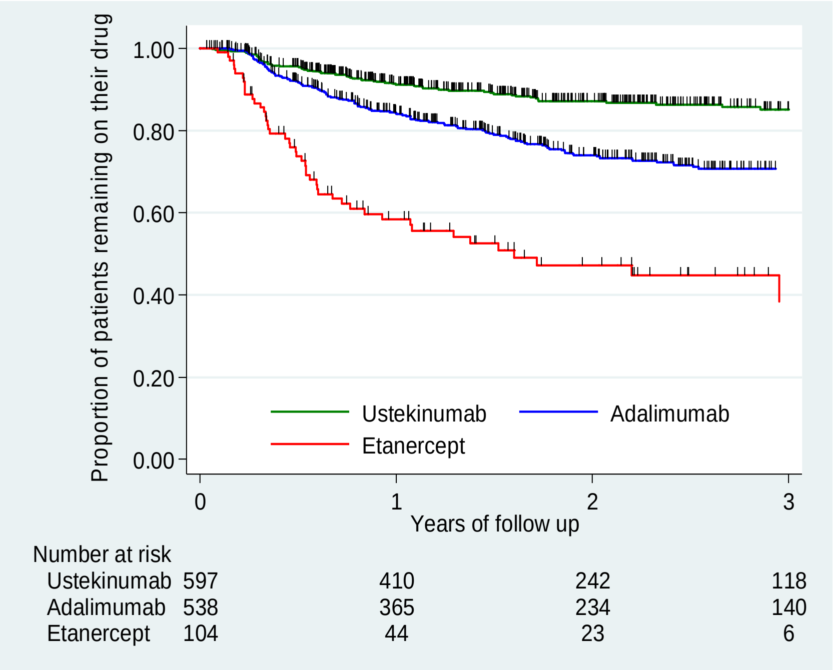

(a) Crude drug survival of the second biologic course limited to discontinuation of the second biologic due to ineffectiveness.

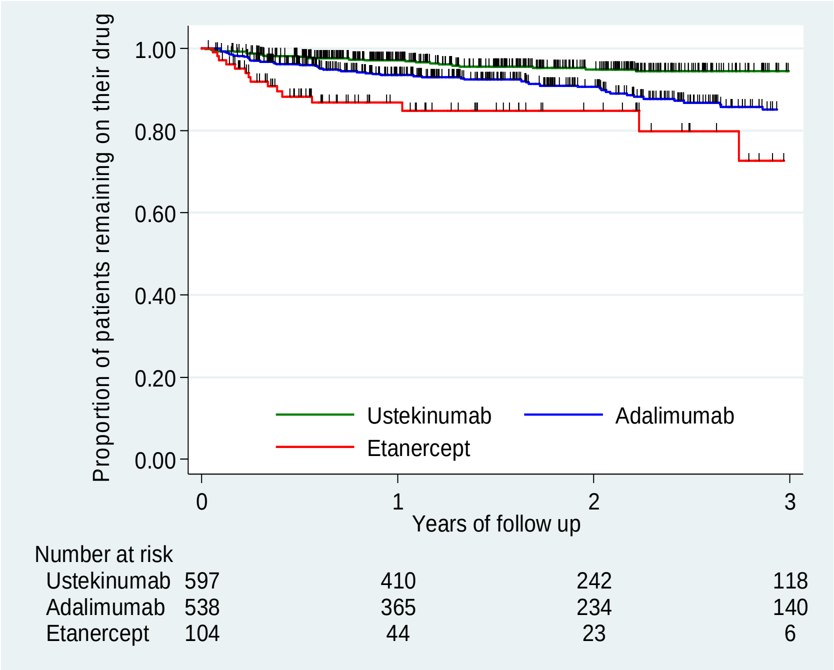

(b) Crude drug survival of the second biologic course limited to discontinuation of the second biologic due to AEs.

Figure S2: Crude drug survival of the second biologic course limited to patients discontinuing first-line etanercept or adalimumab.

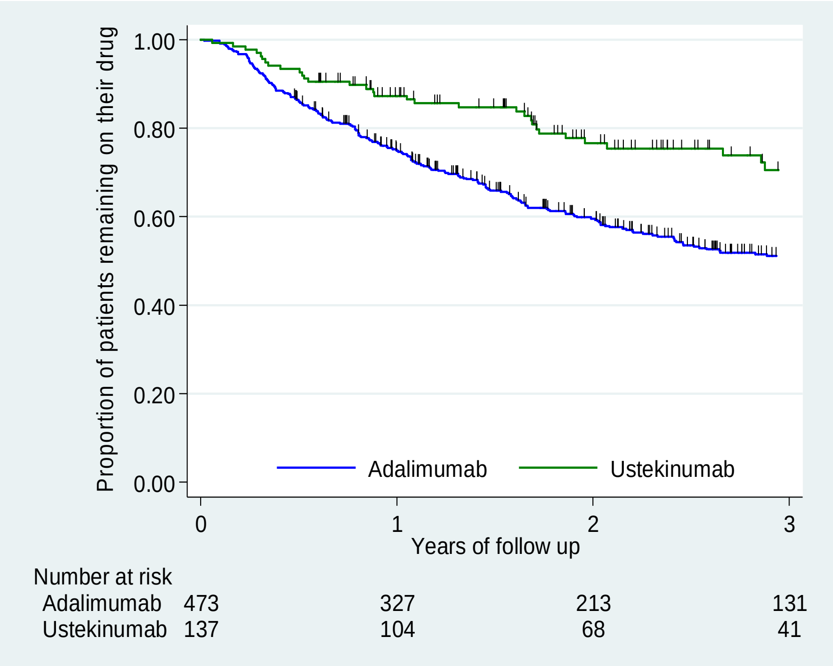

(a) Patients discontinuing first-line etanercept who were switched to second-line adalimumab or ustekinumab

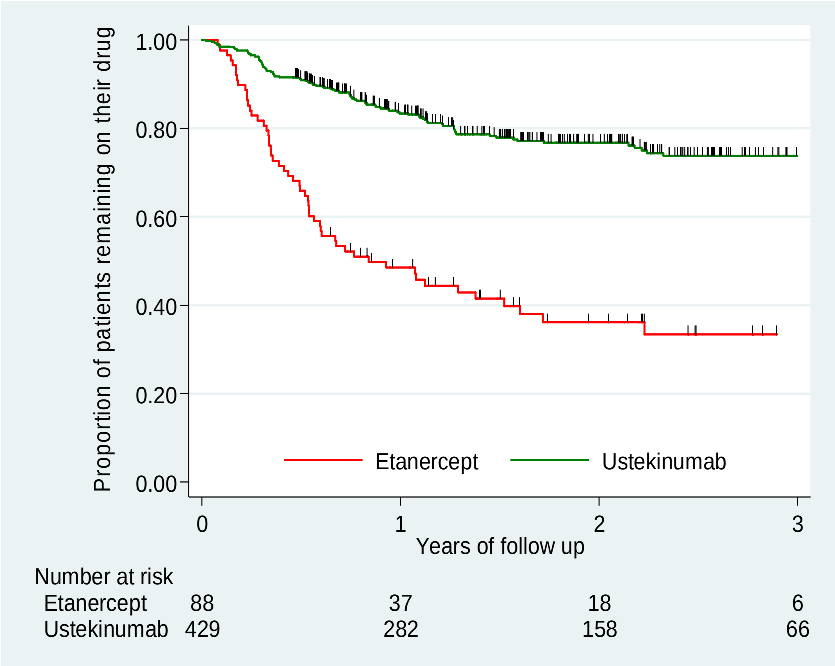

(b) Patients discontinuing first-line adalimumab who were switched to second-line etanercept or ustekinumab
